# Binoxide of Mercury in Skin Diseases

**Published:** 1847-09

**Authors:** 


*Binoxide of Mercury in Skin Diseases.*—M. Ibreisle has reported the results of his experience in the use of the binoxide of mercury as a local application, in diseases of the skin, especially those of a strumous or syphilitic origin. Indolent ulcers of the extremities have frequently yielded to this application, as have also phagedenic ulcerations. The author regards this preparation as an excellent remedy for the removal of the indurations which accompany syphilitic ulcers; he has likewise used it with success in pustular syphilides.

The formula is that of an ointment, consisting of one part of the oxide, to four or five of lead. Some caution appears to be necessary in its employment.—*Ibid, from Gazette Médicale.*

